# Effect of personalized perioperative blood pressure management on postoperative complications and mortality in high-risk patients having major abdominal surgery: protocol for a multicenter randomized trial (IMPROVE-multi)

**DOI:** 10.1186/s13063-022-06854-0

**Published:** 2022-11-17

**Authors:** Alina Bergholz, Agnes S. Meidert, Moritz Flick, Linda Krause, Eik Vettorazzi, Antonia Zapf, Frank M. Brunkhorst, Patrick Meybohm, Kai Zacharowski, Alexander Zarbock, Daniel I. Sessler, Karim Kouz, Bernd Saugel

**Affiliations:** 1 Department of Anesthesiology, Center of Anesthesiology and Intensive Care Medicine, University Medical Center Hamburg-Eppendorf, Martinistrasse 52, 20246 Hamburg, Germany; 2 Department of Anaesthesiology, University Hospital LMU Munich, Munich, Germany; 3 Institute of Medical Biometry and Epidemiology, University Medical Center Hamburg-Eppendorf, Hamburg, Germany; 4 Center for Clinical Studies, Jena University Hospital, Jena, Germany; 5 Center for Sepsis Control and Care, Jena University Hospital, Jena, Germany; 6 Department of Anesthesiology, Intensive Care, Emergency and Pain Medicine, University Hospital Würzburg, Würzburg, Germany; 7 Department of Anaesthesiology, Intensive Care Medicine and Pain Therapy, University Hospital Frankfurt, Goethe University, Frankfurt, Germany; 8 Department of Anesthesiology, Intensive Care, and Pain Medicine, University Hospital Münster, Münster, Germany; 9 Department of Outcomes Research, Anesthesiology Institute, Cleveland Clinic, Cleveland, OH USA; 10 Outcomes Research Consortium, Cleveland, OH USA

**Keywords:** anesthesia, cardiovascular dynamics, hemodynamic monitoring, individualized, randomized controlled trial

## Abstract

**Background:**

Intraoperative hypotension is common in patients having non-cardiac surgery and is associated with serious complications and death. However, optimal intraoperative blood pressures for individual patients remain unknown. We therefore aim to test the hypothesis that personalized perioperative blood pressure management—based on preoperative automated blood pressure monitoring—reduces the incidence of a composite outcome of acute kidney injury, acute myocardial injury, non-fatal cardiac arrest, and death within 7 days after surgery compared to routine blood pressure management in high-risk patients having major abdominal surgery.

**Methods:**

IMPROVE-multi is a multicenter randomized trial in 1272 high-risk patients having elective major abdominal surgery that we plan to conduct at 16 German university medical centers. Preoperative automated blood pressure monitoring using upper arm cuff oscillometry will be performed in all patients for one night to obtain the mean of the nighttime mean arterial pressures. Patients will then be randomized either to personalized blood pressure management or to routine blood pressure management. In patients assigned to personalized management, intraoperative mean arterial pressure will be maintained at least at the mean of the nighttime mean arterial pressures. In patients assigned to routine management, intraoperative blood pressure will be managed per routine. The primary outcome will be a composite of acute kidney injury, acute myocardial injury, non-fatal cardiac arrest, and death within 7 days after surgery.

**Discussion:**

Our trial will determine whether personalized perioperative blood pressure management reduces the incidence of major postoperative complications and death within 7 days after surgery compared to routine blood pressure management in high-risk patients having major abdominal surgery.

**Trial registration:**

ClinicalTrials.gov NCT05416944. Registered on June 14, 2022.

**Supplementary Information:**

The online version contains supplementary material available at 10.1186/s13063-022-06854-0.

## Background

About 2% of patients having inpatient non-cardiac surgery die within the first month after surgery in Europe [1] and the USA [2]. If the first month after surgery would be considered a disease, it would be the third leading cause of death worldwide [3]. Postoperative deaths are a consequence of postoperative complications—including acute kidney injury and acute myocardial injury [2]. To improve postoperative outcomes, modifiable risk factors for complications should be identified and addressed. One of these modifiable risk factors may be intraoperative hypotension.

Intraoperative hypotension is common in patients having non-cardiac surgery and is associated with serious complications—such as postoperative acute kidney and myocardial injury [4–6]—and even death [6, 7]. The association between intraoperative hypotension and serious complications is supported by many observational database studies [8]—but the extent to which the relation is causal remains unclear.

Optimal intraoperative blood pressures for individual patients also remain unknown. On a population basis, intraoperative mean arterial pressures less than 60–70 mmHg are associated with organ injury [4, 5, 9], leading to the recommendation that intraoperative mean arterial pressures should generally be kept above 65 mmHg [10, 11].

Universally targeting higher intraoperative mean arterial pressures (e.g., 75 mmHg vs. 60 mmHg) does not reduce postoperative complications in patients having major non-cardiac surgery [12]. In contrast, the randomized INPRESS trial suggests that individualized blood pressure management targeting preoperative resting blood pressures reduces postoperative complications compared to routine blood pressure management [13]. However, in the INPRESS trial, individual blood pressure targets were defined based on single preoperative blood pressure measurements which do not adequately reflect individual blood pressure profiles [13–16]. Preoperative automated blood pressure monitoring is thought to be the optimal method to establish baseline values before surgery [17]. We thus propose preoperative automated blood pressure monitoring to define individual intraoperative blood pressure targets.

We aim to test the hypothesis that personalized perioperative blood pressure management—based on preoperative automated blood pressure monitoring—reduces the incidence of a composite outcome of acute kidney injury, acute myocardial injury, non-fatal cardiac arrest, and death within 7 days after surgery compared to routine blood pressure management in high-risk patients having major abdominal surgery.

## Methods/design

### Trial design

The proposed trial will be conducted in accordance with the ethical principles based on the Declaration of Helsinki [18] and the ICH E6 R2 guidelines for good clinical practice. The trial was approved by the ethics committee Hamburg (Ethikkommission der Ärztekammer Hamburg, Hamburg, Germany, registration number 2022–100879-BO-ff) acting as the primary ethics committee for this trial. Secondary ethics committee approvals will be obtained for each trial site before patient recruitment starts. The trial was registered at ClinicalTrials.gov (NCT05416944) on June 14, 2022. This article adheres to the Standard Protocol Items: Recommendations for Interventional Trials (SPIRIT) statement (Additional file 1) [19].

IMPROVE-multi will be a multicenter randomized controlled blinded (participating patients, outcome adjudicators, and data analysts) clinical superiority trial in 1272 high-risk patients having elective major abdominal surgery. We plan to enroll patients at approximately 16 German university medical centers. The trial coordinating center will be the University Medical Center Hamburg-Eppendorf, Hamburg, Germany.

### Patients

We will include consenting patients ≥ 45 years scheduled for elective major abdominal surgery with general anesthesia that is expected to last ≥ 90 min. Patients must fulfill at least one of the following high-risk criteria: exercise tolerance < 4 metabolic equivalents as defined by the guidelines of the American College of Cardiology/American Heart Association; renal impairment (serum creatinine ≥ 1.3 mg/dL or estimated glomerular filtration rate < 90 mL/min/1.73 m^2^ within the last 6 months); coronary artery disease (any stage); chronic heart failure (New York Heart Association Functional Classification ≥ II); valvular heart disease (moderate or severe); history of stroke; peripheral arterial occlusive disease (any stage); chronic obstructive pulmonary disease (any stage) or pulmonary fibrosis (any stage); diabetes mellitus requiring oral hypoglycemic agent or insulin; immunodeficiency due to a disease (e.g., HIV, leukemia, multiple myeloma, solid organ cancer) or therapy (e.g., immunosuppressants, chemotherapy, radiation, steroids [above Cushing threshold]); liver cirrhosis (any Child–Pugh class); body mass index ≥ 30 kg/m^2^; current smoking or 15 pack-year history of smoking; age ≥ 65 years; expected anesthesia duration ≥ 180 min; B-type natriuretic peptide > 80 ng/L or N-terminal B-type natriuretic peptide > 200 ng/L within the last 6 months.

We will not include patients having emergency surgery, nephrectomy, and liver or kidney transplantation; patients who had kidney, liver, heart, or lung transplantation; patients having sepsis (according to current Sepsis-3 definition); pregnant women; and patients in whom preoperative automated blood pressure monitoring is not possible.

### Protocol

**Fig. 1:**
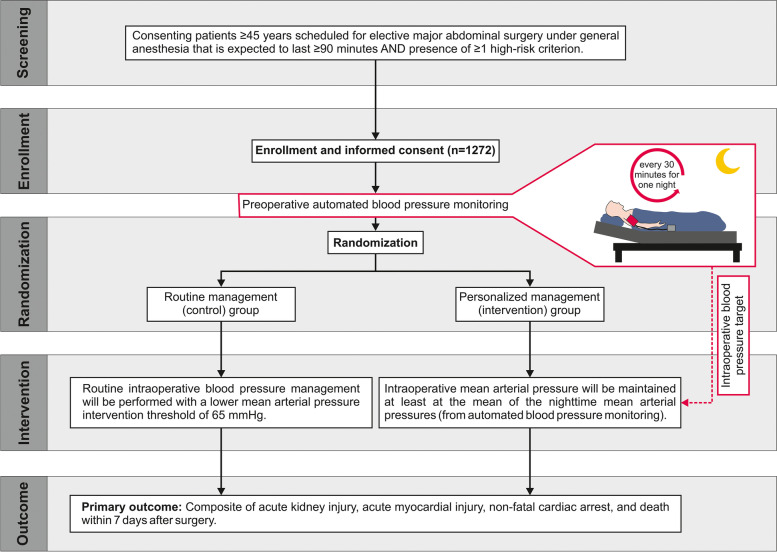
Flowchart illustrating patient screening, enrollment, randomization, trial intervention, and the primary outcome

The trial flow and measures for each participating patient are provided in Fig. 1 and Table 1.

**Table 1:** Frequency and scope of trial visits

	Preoperative	Perioperative	Postoperative						
Day	− *x* to − 1	0	1	2	3	4–6	7	30	90
Visits/contacts	I	II	III	IV	V	VI	VII	VIII	IX
**Informed consent**	X								
**Baseline assessment (i.e., demographic data, medical history)**	X								
**Preoperative automated blood pressure monitoring**	X								
**Blood sampling #1 (baseline values)**	(X)	X							
**Randomization**		X							
**Perioperative blood pressure management**		X							
**Blood sampling #2, 3, 4, and 5**			X	X	X	(X)	X^a^		
**Primary outcome assessment**							X		
**Secondary outcome assessments**					X		X	X	X

^a^If the patient is discharged earlier than day 7, creatinine and high-sensitivity troponin concentrations will be required on the day of hospital discharge

Written informed consent will be obtained by dedicated trial personnel. Baseline demographic and medical characteristics will be recorded and entered in an electronic Case Report Form (eCRF). Preoperative automated blood pressure monitoring will be performed either at home or in the hospital for one night using upper arm cuff oscillometry at 30-min intervals. After excluding artifacts, we will calculate the mean of the nighttime mean arterial pressures. We will define nighttime as 00:00 to 06:00 h [20, 21].

Patients will be randomized in a 1:1 ratio using central block-wise randomization with variable block length stratified by centers with an electronic tool to personalized blood pressure management or to routine blood pressure management. The randomization will be performed directly before the induction of general anesthesia.

In patients assigned to personalized management, intraoperative mean arterial pressure will be maintained at least at the mean of the nighttime mean arterial pressures. If the mean of the nighttime mean arterial pressures will be lower than 65 mmHg, the intraoperative mean arterial pressure will be maintained at least at 65 mmHg; if the mean of the nighttime mean arterial pressures will be higher than 110 mmHg, the intraoperative mean arterial pressure will be maintained only at least at 110 mmHg. The intervention period will start at the induction of general anesthesia and will end 2 h after the end of surgery.

In patients assigned to routine management, intraoperative blood pressure will be managed per routine which usually includes keeping mean arterial pressure ≥ 65 mmHg [5, 10]. Anesthesiologists caring for patients assigned to routine management will not be informed of nighttime blood pressures.

In both groups, the interventions used to achieve the mean arterial pressure target will be at the discretion of treating anesthesiologists and will include intravenous fluid administration, vasoactive medications, adjusting anesthetic depth, and modifying patient position.

In all patients, perioperative blood pressure will be routinely monitored using intraarterial blood pressure monitoring (arterial catheter) or upper arm cuff oscillometry. All other anesthesiologic and surgical procedures will be performed according to routine care and the discretion of the attending anesthesiologists.

### Outcomes

#### Primary outcome

The primary outcome will be a composite of acute kidney injury, acute myocardial injury, non-fatal cardiac arrest, and death within 7 days after surgery.

#### Secondary outcomes

Secondary outcomes will be as follows:

- Incidence of the composite primary outcome within 3 days after surgery and the incidences of each of the individual components of the primary outcome within 3 and 7 days after surgery
- Incidence of a composite outcome of need for renal replacement therapy, myocardial infarction, non-fatal cardiac arrest, and death within 30 and 90 days after surgery and the incidences of each of the individual components of this composite outcome within 30 and 90 days after surgery
- Incidence of a composite outcome of infectious complications within 7 days after surgery and the incidences of each of the individual components of this composite outcome within 7 days after surgery
- Time-to-event outcome with the event “transfer from intensive care unit to normal ward” within 90 days after surgery
- Time-to-event outcome with the event “hospital discharge” within 90 days after surgery
- Incidence of unplanned hospital re-admission within 30 days after surgery

#### Outcome definitions and measures

Acute kidney injury will be defined as an increase in serum creatinine of ≥ 50% from baseline or need for renal replacement therapy within the first 7 postoperative days [22].

Acute myocardial injury will be defined as an increase in high-sensitivity troponin concentration within the first 7 postoperative days according to the definition of “myocardial injury and infarction associated with non-cardiac procedures” set forth in the Fourth Universal Definition of Myocardial Infarction (2018) [23].

Serum creatinine and high-sensitivity troponin I or T (whichever is used in each center) will be measured before surgery (baseline; within the last 30 preoperative days) and on postoperative days 1, 2, 3, and 7. When patients are discharged earlier, serum creatinine and high-sensitivity troponin will be measured on the day of discharge. Additional serum creatinine and high-sensitivity troponin concentrations will be recorded during the initial 30 postoperative days on an as-available basis.

Non-fatal cardiac arrest will be defined as successful resuscitation from ventricular fibrillation, sustained ventricular tachycardia, asystole, or pulseless electrical activity requiring cardiopulmonary resuscitation, pharmacological therapy, or cardiac defibrillation.

Infectious complications will be defined according to the “Standardised Endpoints in Perioperative Medicine (StEP)” initiative [24] and include fever, respiratory infection, neurological infection, urinary system infection, colitis or infection with *Clostridium difficile*, endometritis, surgical site infection, deep incisional surgical site infection, organ or space surgical site infection, unknown infection with pathogenic organisms in tissue or fluid, and sepsis.

Data for the outcome assessment will be collected using medical records and telephone interviews on postoperative days 3, 7, 30, and 90.

Serious adverse events will be documented during the trial and will be reported to the primary ethics committee.

### Statistical considerations and methods

#### Sample size

The sample size was estimated from the anticipated incidence of the composite primary outcome of at least one severe complication or death within 7 days after surgery. We expected an incidence of 25% in the routine management group (adapted from own data and literature [2, 25, 26]) and 17% in the personalized management group. The assumed absolute risk reduction of 8% is a conservative estimate based on a previous trial investigating the effect of individualized blood pressure management compared to routine blood pressure management on postoperative organ failure [13].

We will use a group sequential-design with one unblinded interim analysis using the O’Brien-Fleming spending function [27]. A total sample size of 1144, i.e., 572 patients per group will provide 90% power to detect a difference of 8% between the groups at a significance level of 0.05 using a two-sided test of proportions with continuity correction including an unblinded interim analysis after 50% of patients are recruited. Allowing a total drop-out rate of 10% (assuming an 8% drop-out rate before randomization and 2% after randomization), we plan to recruit 1272 patients. We assume that all patients will remain in their allocated treatment group throughout the observation period. PASS version 16.0.3 was used for the sample size calculation.

#### Statistical analyses

Descriptive analysis will be performed to describe the patient characteristics and clinical data. The primary and secondary outcomes will be analyzed according to the intention-to-treat principle. The primary outcome analyses (interim as well as final analysis) will be conducted using a two-sided test of proportions with continuity correction. When a total of 636 patients will have been observed for 7 days, an unblinded interim analysis will be performed. If the resulting *p*-value is < 0.0015 one-sided, the null hypothesis will be rejected, and the trial will be stopped for efficacy. Otherwise, recruitment will be continued. At the end of the trial, the statistical test for the primary outcome will be performed at a significance level of 0.049 two-sided.

In secondary outcome analyses, the incidence of each of the individual components of the primary outcome and of the additional secondary outcomes will be analyzed using a two-sided test of proportions with continuity correction. In additional analyses, the primary outcome and its individual components will be treated as time-to-event outcomes. Due to the competing event death, all time-to-event outcomes will be assessed using Aalen-Johansen estimators and accompanied by effect estimates based on cause-specific Cox regressions. In further analyses, the primary and secondary outcome analyses will be repeated in the per-protocol population. As a sensitivity analysis, the primary outcome will be analyzed using a mixed logistic regression model including a fixed effect for the random group and a random effect for the center to account for the cluster structure in the data. In further sensitivity analyses, regression models (logistic and cause-specific Cox regression) including potential prognostic baseline variables will be used.

If more than 5% of values are missing for the primary outcome, we will use multiple imputation in a sensitivity analysis. The number of imputations for multiple imputation will be chosen depending on the proportion of missing data according to White et al. [28].

A full statistical analysis plan will be developed before any data are evaluated.

#### Methods against bias

Screening logs will be collected to assess the risk for selection bias in patient recruitment. Randomization codes will be provided by an independent biostatistician to ensure blinding as long as practical. Participating patients, outcome adjudicators, and data analysts will be blinded to group assignment. Furthermore, a blinded outcome committee will be implemented to avoid detection bias. Anesthesiologists treating patients assigned to personalized management cannot be blinded as the results of preoperative automated blood pressure monitoring are necessary for perioperative personalized blood pressure management.

### Data management and monitoring

Patient data will be documented by dedicated trial personnel and managed in an eCRF using a trial management software. Data will be entered via an encrypted connection (HTTPS) in web browser input masks. Each patient will be given an identification number to ensure pseudonymized data analysis. Each change in the data, e.g., due to resolved queries, will be documented by an automated audit trail. Pseudonymized data will be stored in accordance with local data protection laws.

Trial site monitoring will be conducted to ensure that the rights and well-being of patients are protected; that the reported trial data are accurate, complete, and verifiable; and that the conduct of the trial follows the currently approved protocol/amendment(s), with good clinical practice and with applicable regulatory requirements. The monitoring comprises the following tasks: training of the trial personnel prior to trial start (site initiation visit), regular on-site monitoring visits, and a close-out visit at the end of the trial. Central elements of the monitoring are the verification of consent and inclusion criteria, along with trial conduct.

### Data Safety Monitoring Board

Three independent experts—including a biostatistician—will be the members of the Data Safety Monitoring Board (DSMB). DSMB members will meet before trial initiation, 3 months after the inclusion of the first patient, thereafter every 6 months, and after the interim analysis—and more often as they deem necessary or advisable. DSMB members will consider by-group results on a blinded basis. If DSMB members identify safety issues, they will have the prerogative to request unblinded outcomes. DSMB members will advise whether to continue, modify, or stop the trial. DSMB members will act independently from the sponsor and competing interests.

### Dissemination plans

The results of this trial will be published in an international peer-reviewed medical journal. Both positive and negative results will be reported. The principal investigator is responsible for the preparation of the final report and therefore will have access to all available data. The full protocol, anonymized data, and the statistical code used for the analysis will be available from the corresponding author upon reasonable request. Authorships will be based on the recommendations of the International Committee of Medical Journal Editors (http://www.icmje.org/).

## Discussion

In this trial, we will test whether personalized perioperative blood pressure management—based on preoperative automated blood pressure monitoring—reduces the incidence of a composite outcome of acute kidney injury, acute myocardial injury, non-fatal cardiac arrest, and death within 7 days after surgery compared to routine blood pressure management in high-risk patients having major abdominal surgery.

At some level, intraoperative hypotension surely causes organ injury—but the harm threshold for individual patients remains unclear. Perioperative blood pressure targets are thus a matter of current research. In a single-center trial including 451 high-risk patients having major non-cardiac surgery, universally targeting mean arterial pressure ≥ 75 mmHg—compared to ≥ 60 mmHg—did not reduce postoperative major adverse cardiovascular events [12]. Interestingly, the authors of this trial conclude that we should move away from population-based to individualized definitions of hypotension, “which could open the door to a paradigm of personalized intraoperative blood pressure targets” [12]. Using a population harm threshold of 65 mmHg or universally targeting higher pressures for all patients indeed ignores the fact that normal blood pressure varies considerably among individuals [15]. From a physiologic perspective, it is thus reasonable to assume that optimal intraoperative blood pressure depends on each individual patient’s normal blood pressure physiology.

There is only one randomized trial on individualized blood pressure management [13]. The INPRESS trial tested the hypothesis that targeting individual preoperative resting blood pressures reduces the incidence of postoperative systemic inflammation and organ dysfunction within 1 week after surgery compared to routine blood pressure management in 292 high-risk patients having major surgery [13]. Individualized blood pressure management reduced the risk of postoperative systemic inflammation and organ dysfunction compared to routine blood pressure management [13]. However, this small trial had limitations that limit its internal validity. First, this trial not only compared two different blood pressure management strategies but also used two different vasopressors (norepinephrine vs. ephedrine) in study and control group patients. Second, the individual intraoperative blood pressure target was defined based on a single preoperative blood pressure measurement taken in the preoperative evaluation clinic or on the surgical ward the day before surgery. However, single preoperative blood pressure measurements do not adequately reflect individual blood pressure profiles [14–16]. We, therefore, will perform preoperative automated blood pressure monitoring for one night to define individual intraoperative blood pressure targets.

Automated blood pressure monitoring is the clinical reference method to assess blood pressure profiles [29] and an international consensus group recently defined it as “the optimal method to establish baseline values” before surgery [17]. We will define nighttime as 00:00 to 06:00 h [20, 21] and thus exclude retiring and rising periods (evening 21:00 to 00:00 h; morning 06:00 to 09:00 h) which are subject to considerable variation [20, 21].

We will include high-risk patients having major abdominal surgery to ensure that the studied population is broad and representative of the patient population benefiting from hypotension avoidance [30]. Participating trial centers will be mainly large university medical centers with many high-risk patients having different types of major abdominal surgery procedures.

Our chosen outcomes are clinically relevant and are recommended by an expert consensus panel of the StEP initiative [31, 32]. Standardized and precisely defined outcomes ensure valid comparisons between different trials [33]. The primary composite outcome includes major perfusion-related complications within 7 days after surgery. Considering complications within the first 7 postoperative days minimizes the risk of complications being confounded by postoperative events rather than intraoperative blood pressure management. In addition, most patients are still hospitalized during the first 7 postoperative days, which allows a more accurate outcome assessment. We will also assess the incidence of a composite outcome of need for renal replacement therapy, myocardial infarction, non-fatal cardiac arrest, and death within 30 and 90 days after surgery.

In summary, we will perform a randomized trial to test the hypothesis that personalized perioperative blood pressure management—based on preoperative automated blood pressure monitoring—reduces the incidence of a composite outcome of acute kidney injury, acute myocardial injury, non-fatal cardiac arrest, and death within 7 days after surgery compared to routine blood pressure management in high-risk patients having major abdominal surgery.

## Trials status

Patient recruitment for this trial is scheduled to start in November 2022 and is estimated to be completed in May 2024. This article is based on the most recent version of the study protocol (i.e., version 1.0, July 2022).

## Supplementary Information

**Additional file 1.** SPIRIT checklist.

## Data Availability

Not applicable.

## Abbreviations

DSMB: Data Safety Monitoring Board
eCRF: electronic Case Report Form
SPIRIT: Standard Protocol Items: Recommendations for Interventional Trials
StEP: Standardised Endpoints in Perioperative Medicine
